# Blossoms amid drought: a bibliometric mapping of research on drought stress in ornamental plants (1995–2025)

**DOI:** 10.3389/fpls.2025.1644092

**Published:** 2025-09-17

**Authors:** Ümmü Özgül Karagüzel

**Affiliations:** Department of Horticulture, Faculty of Agriculture, Recep Tayyip Erdoğan University, Rize, Türkiye

**Keywords:** drought stress, floriculture, physiological responses, omics, climate-resilient horticulture, collaboration networks flowering

## Abstract

Drought stress is a major abiotic constraint limiting plant growth and ornamental quality. Despite the importance of ornamental species in global horticulture, they remain underrepresented in drought-related research compared to food and industrial crops. This study presents a bibliometric and network-based analysis of drought stress research in ornamentals from 1995 to 2025, based on 1,387 records from Web of Science and 1,212 from Scopus. After screening, 383 WoS and 436 Scopus records were retained, yielding 819 articles. Keyword analysis showed dominant themes in gas exchange, photosynthesis, stomatal conductance, proline, and antioxidant activity. Recent inclusion of transcription factors, RNA-seq, and proteomics suggests a growing molecular focus. Salt tolerance, evapotranspiration, and floral traits under drought were also highlighted. Microbial strategies, such as PGPR and mycorrhizae, appeared infrequently. China, the USA, and Spain were the leading contributors, supported by international collaborations. Core journals included HortScience, Scientia Horticulturae and Frontiers in Plant Science. This work outlines the field’s thematic structure and evolution, underscoring the need to integrate physiological, molecular, and ecological tools to strengthen drought resilience in ornamentals. This study conducts a longitudinal and network-based bibliometric analysis of drought stress research in ornamental plants, drawing from peer-reviewed literature published between 1995 and 2025 across the Web of Science Core Collection and Scopus databases.

## 1 Introduction

**Figure 1:**
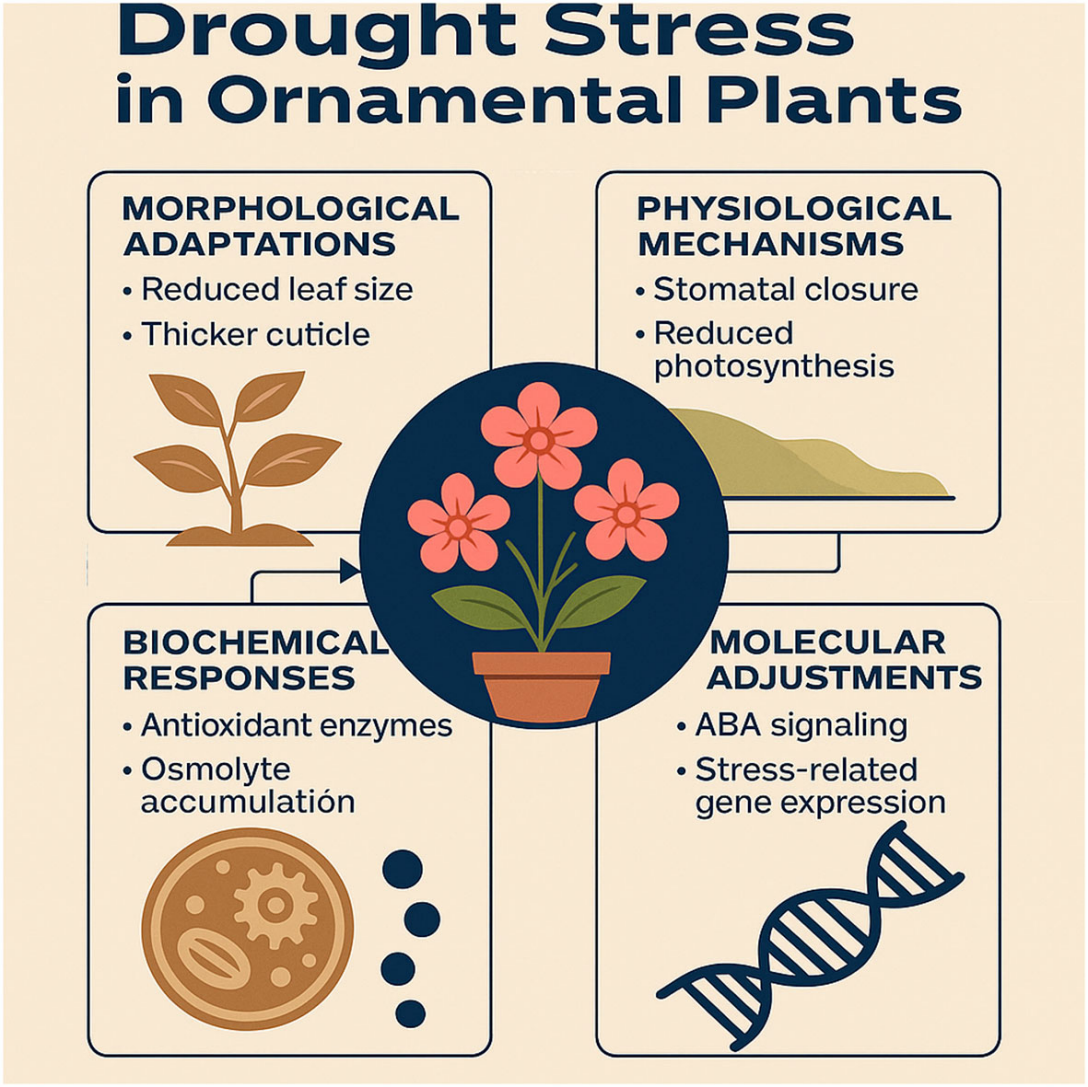
Key mechanisms underlying drought adaptation in ornamental horticulture.

Drought is among the most critical abiotic stressors threatening global horticulture, imposing severe constraints on plant growth by limiting water uptake, altering metabolism, and destabilizing physiological processes (Anjum et al., 2011; Kapoor et al., 2020). Projections from the IPCC Sixth Assessment Report (2021) indicate that global temperatures may rise by up to 4.4°C by 2100 under high-emission scenarios, further increasing drought frequency and severity worldwide (Molina et al., 2025). While its impacts on staple crops are extensively documented, drought effects on ornamental plants despite their high aesthetic and economic value remain underexplored. In ornamental horticulture, drought can sharply reduce market value by lowering floral quality and foliage aesthetics, causing premature aging, reduced flowering, shorter stems, smaller leaves, and lower biomass (Toscano et al., 2019; Sánchez-Blanco et al., 2019; Rebi et al., 2024; Zhao et al., 2024; Chachar et al., 2025). Early detection remains difficult, but markers such as proline, malondialdehyde, antioxidant enzyme activity, and chlorophyll fluorescence are reliable measures of stress intensity (Toscano et al., 2016; Jafari et al., 2019; Toscano and Romano, 2021; Cui et al., 2022; Kittipornkul et al., 2024) and provide insights into adaptation mechanisms (Mircea et al., 2024). Morphological traits (reduced leaf size, thicker cuticle) minimize water loss; physiological responses (stomatal closure, reduced photosynthesis) improve water use; biochemical defenses involve antioxidants and osmolytes; and molecular adjustments include ABA signaling and stress-related gene expression (Toscano et al., 2016). Omics approaches transcriptomics, metabolomics, proteomics—have advanced understanding of these responses in ornamentals (Zhang et al., 2021a, b; Sahithi et al., 2021; Xiong et al., 2021; Cui et al., 2023; Naresh et al., 2024) (**Figure 1**). Bibliometric tools now map research trends and thematic evolution, yet analyses on ornamentals are rare.

This study aims to systematically examine global research output on drought stress in ornamental plants between 1995 and 2025 using bibliometric and scientometric approaches. It explores temporal trends and growth dynamics in scientific production, identifies the most influential countries, institutions, and authors contributing to the field, and analyzes recurring keywords and thematic clusters to illustrate the conceptual structure of research. Furthermore, it highlights critical knowledge gaps, neglected topics, and emerging research fronts, providing a comprehensive foundation for guiding future studies aimed at enhancing drought tolerance in ornamental horticulture.

## 2 Methods

### 2.1 Review framework and PICOS strategy

The research question guiding this systematic mapping review was structured according to the PICOS framework, adapted to the context of bibliometric analysis:

Population (P): Peer-reviewed scientific publications related to drought stress in ornamental plants published between 1995 and 2025.
Intervention (I): Application of bibliometric tools and network visualization methods to assess research trends and patterns.
Comparators (C): Not applicable, as the study does not involve experimental groups or comparative treatments.
Outcomes (O): Identification of publication trends, prolific authors, institutional and international collaboration, core journals, and evolving research themes.
Study Design (S): Systematic mapping review based on data retrieved from Scopus and Web of Science databases using predefined inclusion criteria and search strategy.

### 2.2 Data sources and search strategy

**Figure 2:**
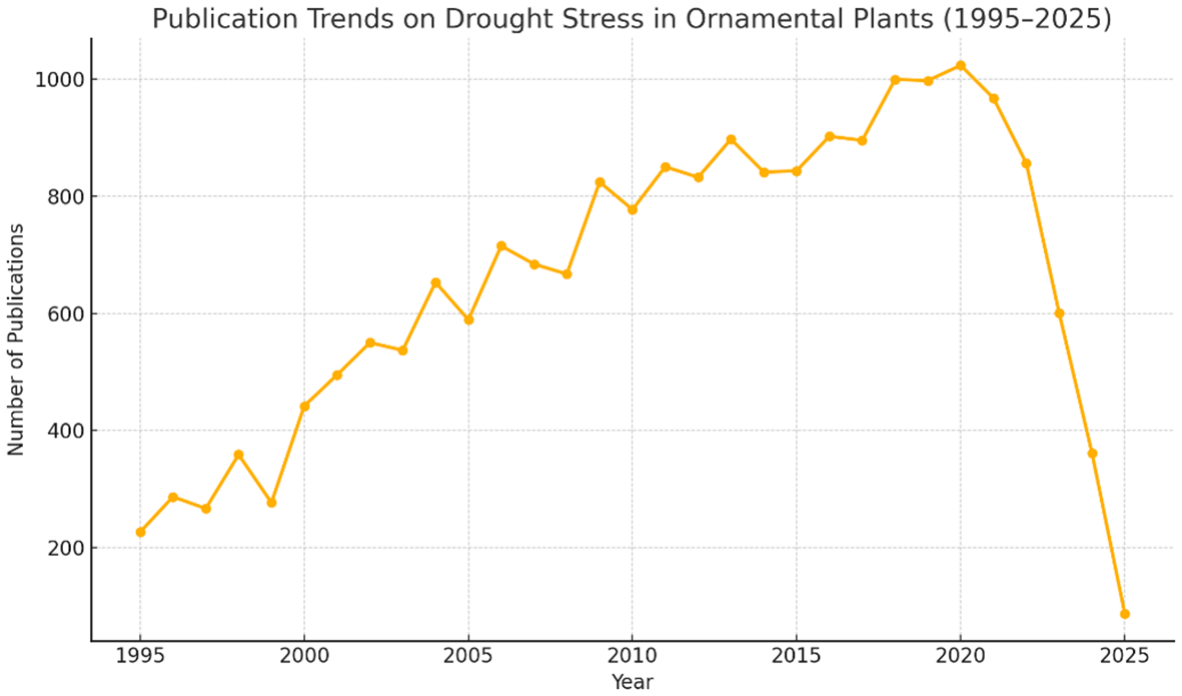
Yearly scientific output and indexing artifacts (1995–2025). Created by the authors using Scopus/WoS data.

Given their broad coverage and credibility within the academic community, the Web of Science Core Collection and Scopus databases were selected to retrieve bibliometric data relevant to the field. The search was conducted in May 2025 and included peer-reviewed articles and review papers published between January 1, 1995, and April 30, 2025. To ensure consistency and comprehensiveness in data collection, the following Boolean query was employed across both databases: (“drought stress” OR “water stress” OR “water deficit” OR “drought tolerance” OR “heat stress” OR “deficit irrigation” OR “restricted irrigation”) AND (“ornamental plants” OR “ornamentals” OR “floriculture”). In the Web of Science, the query was applied using the Topic Search (TS) field, which encompasses the title, abstract, author keywords, and Keywords Plus. In Scopus, the same query was executed across the title, abstract, and keyword fields. No restrictions were imposed on language, document type, or indexing category to preserve the inclusivity of the dataset. The retrieved records were screened manually to determine thematic relevance. Titles, abstracts, and keywords were carefully examined, and publications unrelated to drought or ornamental species were excluded. This process specifically removed studies focusing on drought stress in non-ornamental crops, forestry species, or non-plant subjects, even if they matched the search terms. The discrepancy in record counts between Scopus (436) and Web of Science (383) can be attributed to differences in database coverage, indexing scope, journal inclusion policies, subject categorization, and keyword-matching algorithms. Notably, Scopus indexes a broader range of horticultural and regionally focused journals, which likely explains part of the higher count. It should be noted that, while the study is designed to cover the period up to 2025, the data extraction date (May 2025) means that publications from the remainder of the year are not fully represented. This temporal limitation is particularly relevant for recently accepted articles not yet indexed at the time of data retrieval and may result in a slight underrepresentation of the most current research trends. The sharp decrease observed between 2024 and 2025 in **Figure 2** is therefore likely attributable to indexing delays in the databases rather than a true decline in research output.

**Figure 3:**
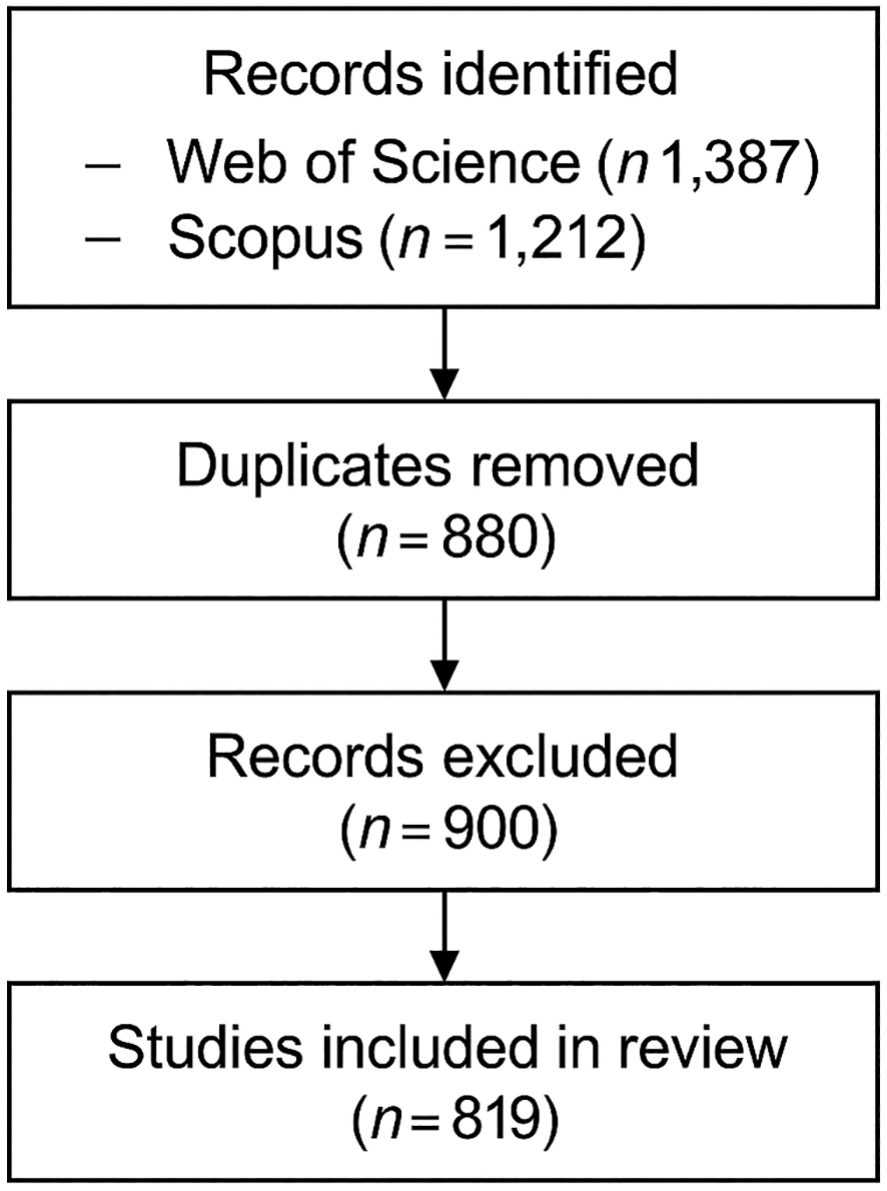
PRISMA 2020 flow diagram showing the identification, screening, and inclusion process of the studies (Page et al., 2021).

The study selection procedure followed the PRISMA 2020 guidelines for systematic reviews (Page et al., 2021), and the process is detailed in the flow diagram (**Figure 3**). The initial search yielded a total of 2,599 records 1,387 from Web of Science and 1,212 from Scopus. Following the removal of 880 duplicates, 900 records were excluded after screening titles and abstracts based on predefined criteria. Specifically, studies were retained only if they focused on drought stress in ornamental plants. Publications addressing drought stress in non-ornamental crops (e.g., cereals, vegetables, fruits, medicinal and aromatic plants), forestry species, or non-plant subjects were excluded, even when search terms matched. Additional inclusion criteria comprised publication type (articles and reviews) and language (English). The final dataset consisted of 819 publications, which formed the basis of the bibliometric mapping. The PRISMA diagram outlines each step of the identification, screening, eligibility, and inclusion phases, ensuring transparency and reproducibility in the data selection process.

### 2.3 Data merging and preprocessing

The bibliographic records obtained from Web of Science (in “plain text” format) and Scopus (in “csv” format) were initially pre-processed to ensure compatibility with VOSviewer (version 1.6.15). Manual adjustments were performed to harmonize metadata structures, particularly field labels corresponding to authors, sources, and keywords. Data cleaning and standardization procedures were carried out using Microsoft Excel, which facilitated the removal of duplicate entries, correction of syntactic inconsistencies, and normalization of metadata across both datasets. This process ensured consistency and reliability in the unified dataset. Subsequently, the cleaned files were merged using the *Create Map Based on Bibliographic Data* function in VOSviewer, with the multiple file input option enabled.

### 2.4 Data analysis and visualization

The integrated bibliographic dataset, compiled from Web of Science and Scopus, was analyzed using VOSviewer (version 1.6.15), a widely used software for constructing and visualizing bibliometric networks. Following data refinement, the bibliographic records from both sources were combined in VOSviewer using the multi-file input feature under the “Create Map Based on Bibliographic Data” option.

### 2.5 Systematic review protocol

This systematic review was not registered in advance and did not follow a pre-specified protocol. However, all methods, including the search strategy, inclusion criteria, and screening process, were transparently documented and strictly adhered to in accordance with PRISMA 2020 guidelines.

### 2.6 Bibliometric mapping and visualization

Using the unified dataset, bibliometric mapping was performed to reveal the thematic organization and collaborative relationships in the field.

Co-authorship networks (authors, countries, institutions)
Citation networks (authors, documents, journals, countries)
Keyword co-occurrence maps (thematic clustering)
Source bibliographic coupling

Each analysis was conducted using minimum occurrence thresholds (e.g., minimum number of documents or citations ≥1), and clustering was based on total link strength. Visual outputs were exported as high-resolution figures for interpretation.

These analyses enabled the identification of leading contributors, dominant themes, and global collaboration patterns in drought stress research related to ornamental plants. **Figure 3** provides a visual summary of both the selection methodology and the analytical structure adopted in this study.

### 2.7 VOSviewer parameter configuration

All bibliometric network analyses were conducted using VOSviewer version 1.6.20 (Leiden University, The Netherlands). Separate analyses were performed for co-authorship (authors, countries, institutions), keyword co-occurrence, and citation/bibliographic coupling. Full counting was applied unless otherwise indicated. The association strength method was used for normalization across all analyses. The LinLog/modularity optimization layout algorithm was applied with attraction = 2 and repulsion = 0 to optimize network clarity. Label size scaling was set to 150% to enhance readability, and clustering resolution was maintained at 1.00. Minimum threshold values for inclusion were determined based on network density and visualization clarity, as summarized in **Table 1**.

**Table 1:** VOSviewer parameter configuration for different bibliometric analyses.

Analysis type	Unit of analysis	Counting method	Threshold (Min.)	Normalization method	Layout algorithm/ parameters	Label size scaling	Clustering resolution/ visualization type
Co-authorship	Authors	Full counting	≥ 3 documents, ≥ 30 citations	Association strength	LinLog, attraction = 2, repulsion = 0	150%	1.00 / Network visualization
Co-authorship	Countries	Full counting	≥ 5 documents	Association strength	LinLog, attraction = 2, repulsion = 0	150%	1.00 / Network visualization
Co-authorship	Institutions	Full counting	≥ 3 documents	Association strength	LinLog, attraction = 2, repulsion = 0	150%	1.00 / Network visualization
Keyword co-occurrence	All keywords	Full counting	≥ 5 occurrences	Association strength	LinLog, attraction = 2, repulsion = 0	150%	1.00 / Overlay visualization
Citation network	Authors	Full counting	≥ 30 citations	Association strength	LinLog, attraction = 2, repulsion = 0	150%	1.00 / Network visualization
Bibliographic coupling	Documents	Full counting	≥ 5 citations	Association strength	LinLog, attraction = 2, repulsion = 0	150%	1.00 / Network visualization

Unit of analysis = network entity (authors, countries, institutions, keywords, or documents). Threshold (Min.) = minimum inclusion; e.g., “≥3 documents, ≥30 citations” means an author must meet both criteria to be included. This was applied to exclude weak nodes and improve clarity. Full counting = each publication counted once per author/country/institution. Association strength = normalization method. LinLog (attraction=2, repulsion=0) = layout algorithm. Label size scaling (150%) = enlarged for readability. Clustering resolution (1.00) = modularity parameter. Visualization type = network maps for authors/journals; overlay map for keywords.

## 3 Results on drought stress trends in ornamental horticulture

### 3.1 Publication trends in drought stress research on ornamental plants

Core bibliometric indicators, such as the total number of publications and their citation frequencies, serve as essential metrics for assessing research productivity and influence. As depicted in **Figure 2**, the number of publications exhibited a steady upward trend over the past three decades, rising from fewer than 250 articles in 1995 to a peak of over 1,000 in 2020. This sustained growth reflects an increasing global concern for water scarcity and its effects on the aesthetic and physiological performance of ornamental species. Following the peak period between 2020 and 2021, a marked decline in annual publication numbers is observed, particularly in 2024 and 2025. This sharp drop is likely due to database indexing delays, which typically affect the completeness of recent-year records in both Scopus and Web of Science. Nonetheless, the overall pattern indicates a long-term expansion of research efforts targeting drought-induced stress mechanisms and management strategies in ornamental horticulture.

Beyond temporal trends, a species-level assessment revealed the taxa that have received the most research attention over the past 30 years (**Table 2**). *Petunia hybrida* emerged as the most frequently studied ornamental species under drought stress, representing 12.59% of all publications. This was followed by *Chrysanthemum morifolium* (8.54%), *Rosa chinensis* (6.71%), *Tagetes erecta* (5.32%), and *Pelargonium × hortorum* (4.91%). The prevalence of these taxa reflects both their global horticultural importance and their commercial relevance in the ornamental plant industry. Conversely, a substantial number of horticulturally valuable species remain underrepresented in the literature, highlighting potential opportunities for future research to broaden the taxonomic scope of drought-resilience studies.

**Table 2:** Most Frequently studied ornamental plant species under drought stress (1995–2025) Based on combined scopus and web of science data.

Species (binomial)	Publications (n)	Share of total (%)
*Petunia hybrida*	18	12.59
*Zinnia elegans*	15	10.49
*Impatiens walleriana*	14	9.79
*Tagetes erecta*	14	9.79
*Cyclamen persicum*	13	9.09
*Chrysanthemum morifolium*	10	6.99
*Catharanthus roseus*	10	6.99
*Rosa chinensis*	8	5.59
*Helianthus annuus*	7	4.9
*Gerbera jamesonii*	7	4.9

### 3.2 Keyword co-occurrence themes in drought stress research

**Figure 4:**
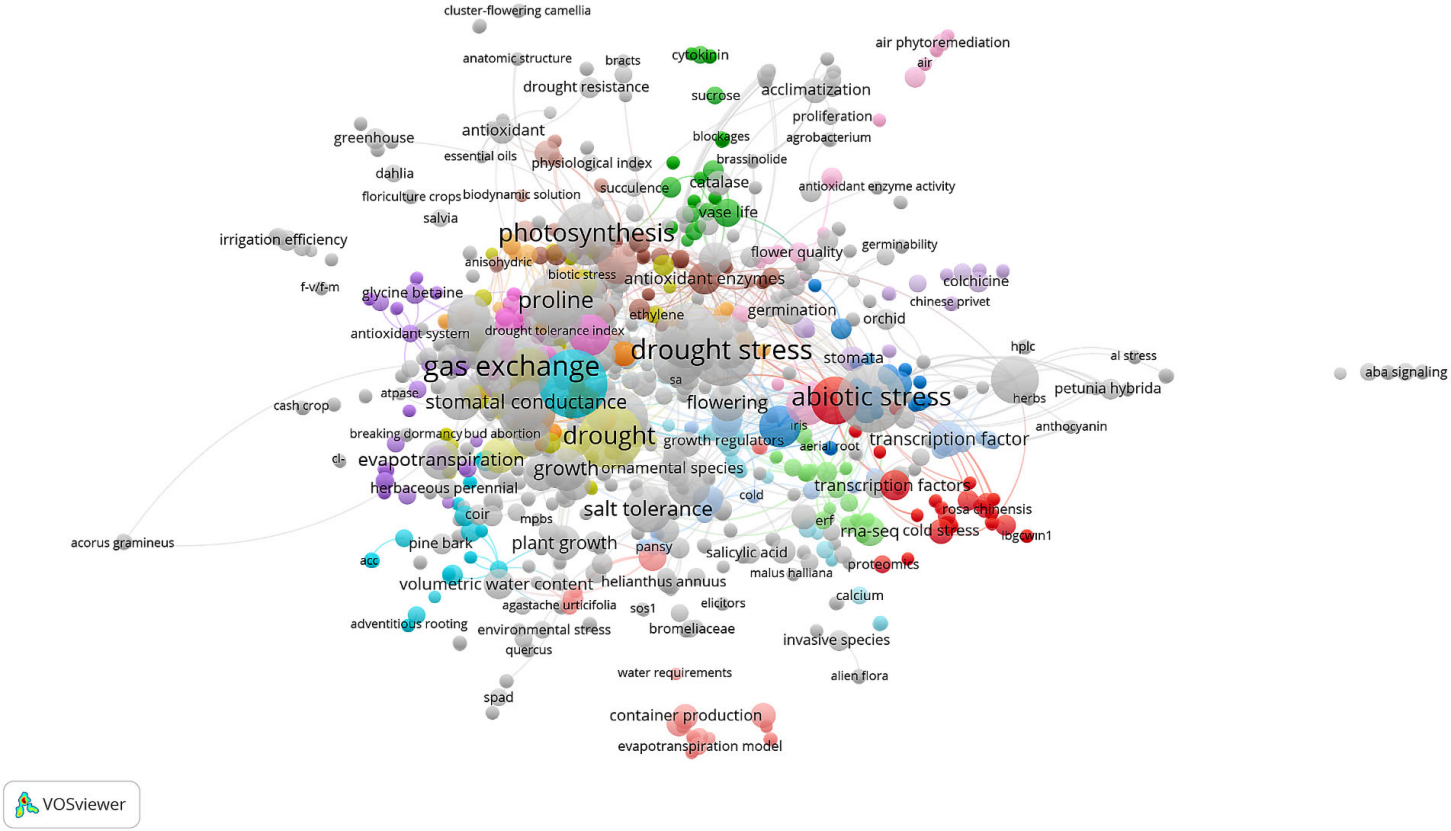
Keyword co-occurrence network (1995–2025). Created by the authors using VOSviewer. Node size represents term frequency; color indicates thematic clusters.

The keyword co-occurrence network shown in **Figure 4** reveals the thematic contours of drought stress studies in ornamental horticulture, with densely grouped terms reflecting distinct and recurring research focal points. At the core of the network, terms such as “drought stress”, “gas exchange”, “photosynthesis”, and “abiotic stress” appear as high-frequency nodes. These keywords form the central axis of research, highlighting the dominant focus on physiological responses to drought conditions. Surrounding clusters reflect specialized thematic areas. One prominent cluster includes terms such as “proline”, “antioxidant enzymes”, and “oxidative stress”, indicating strong interest in the biochemical defense mechanisms of plants under drought. Another major cluster is defined by terms like “transcription factors”, “gene expression”, and “ma-seq”, pointing to the integration of molecular and transcriptomic approaches in more recent publications. Additional peripheral themes include evapotranspiration models, irrigation efficiency, flower quality, and root development, which reflect applied research directions targeting water management and plant performance optimization in ornamental crops. The network structure reveals a mature research domain, enriched by the integration of classical physiological knowledge with emerging molecular techniques and practical applications. This thematic diversity signals sustained growth and innovation.

### 3.3 Leading journals in drought stress and ornamentals research

**Figure 5:**
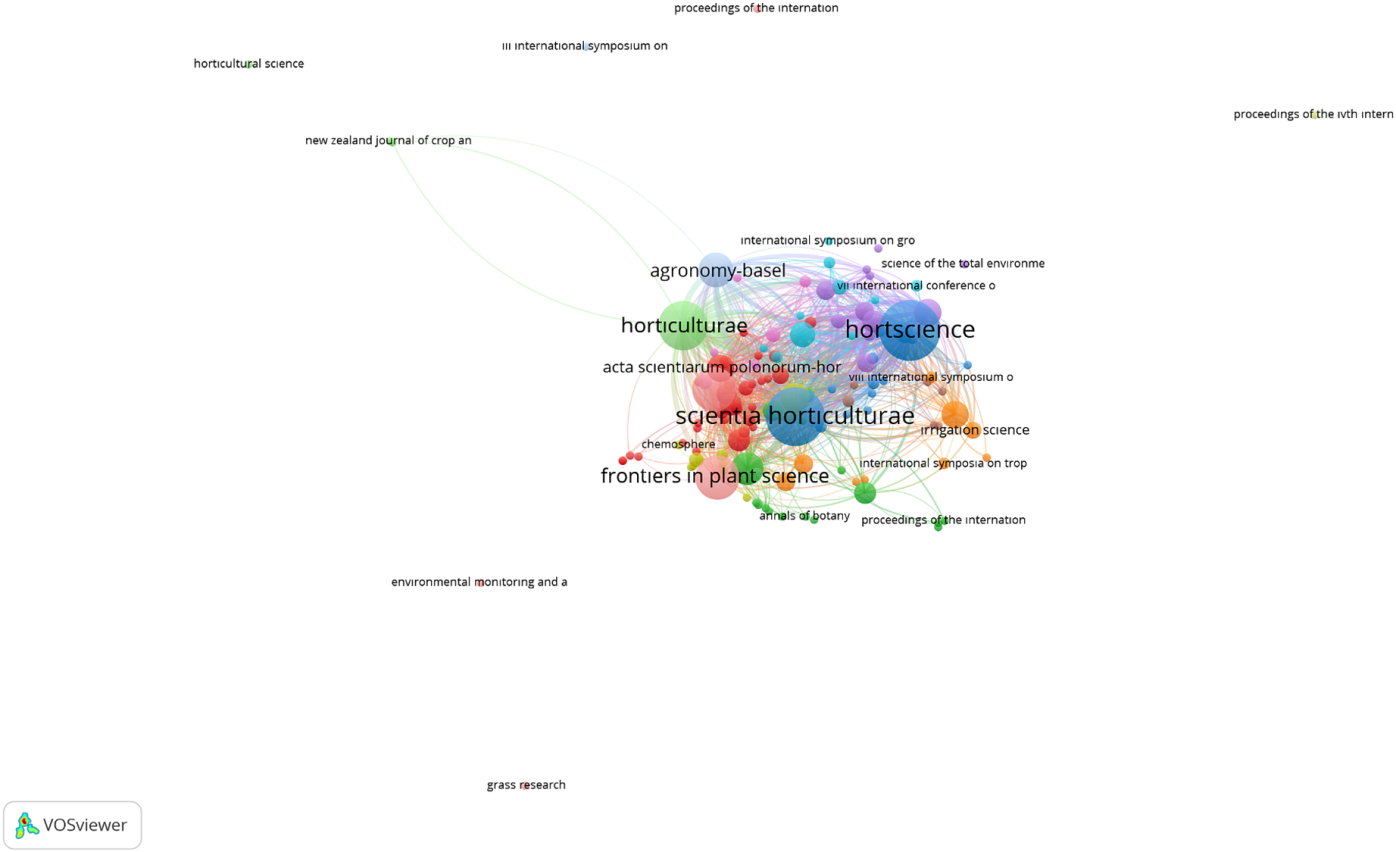
Journal bibliographic coupling network (1995–2025). Created by the authors using VOSviewer. Node size reflects publication volume; proximity and links indicate thematic similarity through shared citations.

The bibliographic coupling map of journals presented in **Figure 5** identifies the core publication sources contributing to drought stress research in ornamental plants. The clustering of journals in this network is driven by overlapping citation patterns, where closer and more strongly linked journals tend to address similar topics and cite comparable sources. Prominent nodes such as Scientia Horticulturae, HortScience, and Frontiers in Plant Science appear at the center of the network, indicating their dominant roles as core dissemination platforms. These journals are characterized by a high volume of publications and strong coupling with multiple other sources, highlighting their centrality in the field’s knowledge structure. Other tightly coupled journals include Agronomy-Basel, Horticulturae, and Acta Horticulturae, which are thematically aligned with applied and empirical studies in horticultural science, particularly under abiotic stress conditions. The presence of Environmental Monitoring and Assessment and New Zealand Journal of Crop and Horticultural Science on the periphery suggests narrower or more region-specific thematic scope with limited cross-journal referencing. The coupling density and clustering pattern indicate that drought stress research in ornamentals is concentrated within a relatively cohesive group of journals, bridging foundational plant science, applied horticulture, and environmental stress disciplines. The network structure offers a valuable reference for researchers seeking to publish in journals with higher thematic coherence and visibility in this domain.

### 3.4 Author collaboration patterns in drought stress research

**Figure 6:**
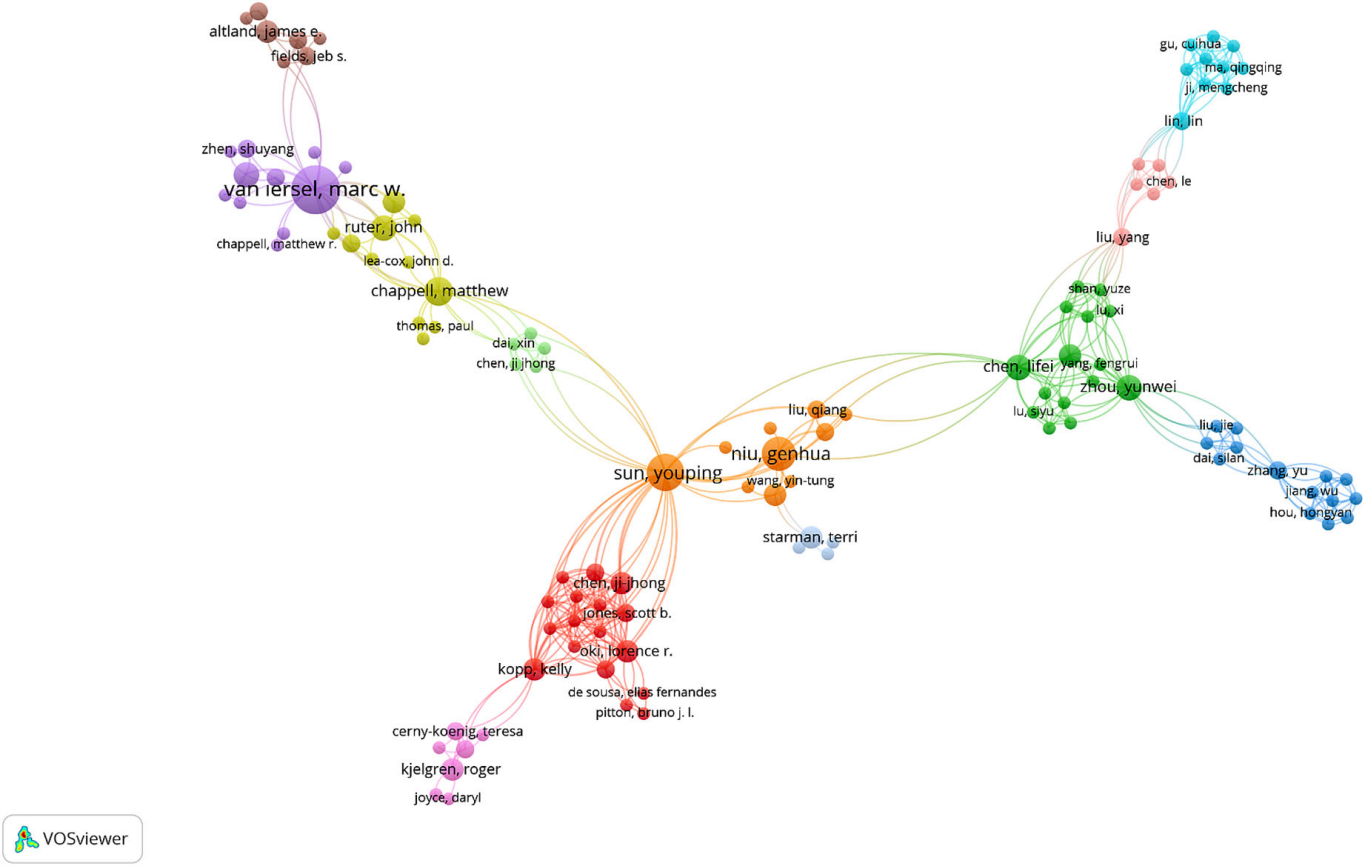
Co-authorship network in drought stress research (1995–2025). Created by the authors using VOSviewer. Node size indicates author productivity; links represent co-authorship strength.

The co-authorship network presented in **Figure 6** reveals the structural dynamics of author collaborations in the field of drought stress and ornamental plants. The visualization identifies several well-defined clusters, each representing distinct research communities that frequently collaborate on publications within this domain. The most prominent node, Sun Youping, appears as a central hub connecting multiple author groups, indicating a high level of productivity and cross-group collaboration. Surrounding this node are secondary clusters led by Niu Genhua and Kopittke Peter M., whose co-authorship patterns suggest stable, long-term collaborations often confined within their institutional or regional affiliations. Other notable clusters include van Iersel Marc W. and Chen Jie, both of whom anchor independent author networks with relatively strong intra-group cohesion but limited cross-linkages to other clusters. This structural feature may reflect thematic specialization or geographic concentration in collaborative practices. The overall structure of the network of the network exhibits a combination of centralized and clustered traits, where a few prolific researchers serve as bridges between otherwise discrete author groups. Such a configuration supports efficient information flow across subfields, while still maintaining specialized research foci within individual clusters. The analysis reveals both integrative and fragmented aspects of current research collaboration. While certain researchers contribute to broader academic cohesion, some clusters remain isolated. Promoting stronger ties across regions and institutions may drive more innovative and diverse approaches in upcoming research on drought stress in ornamentals.

### 3.5 Institutional networks in ornamental plant drought research

**Figure 7:**
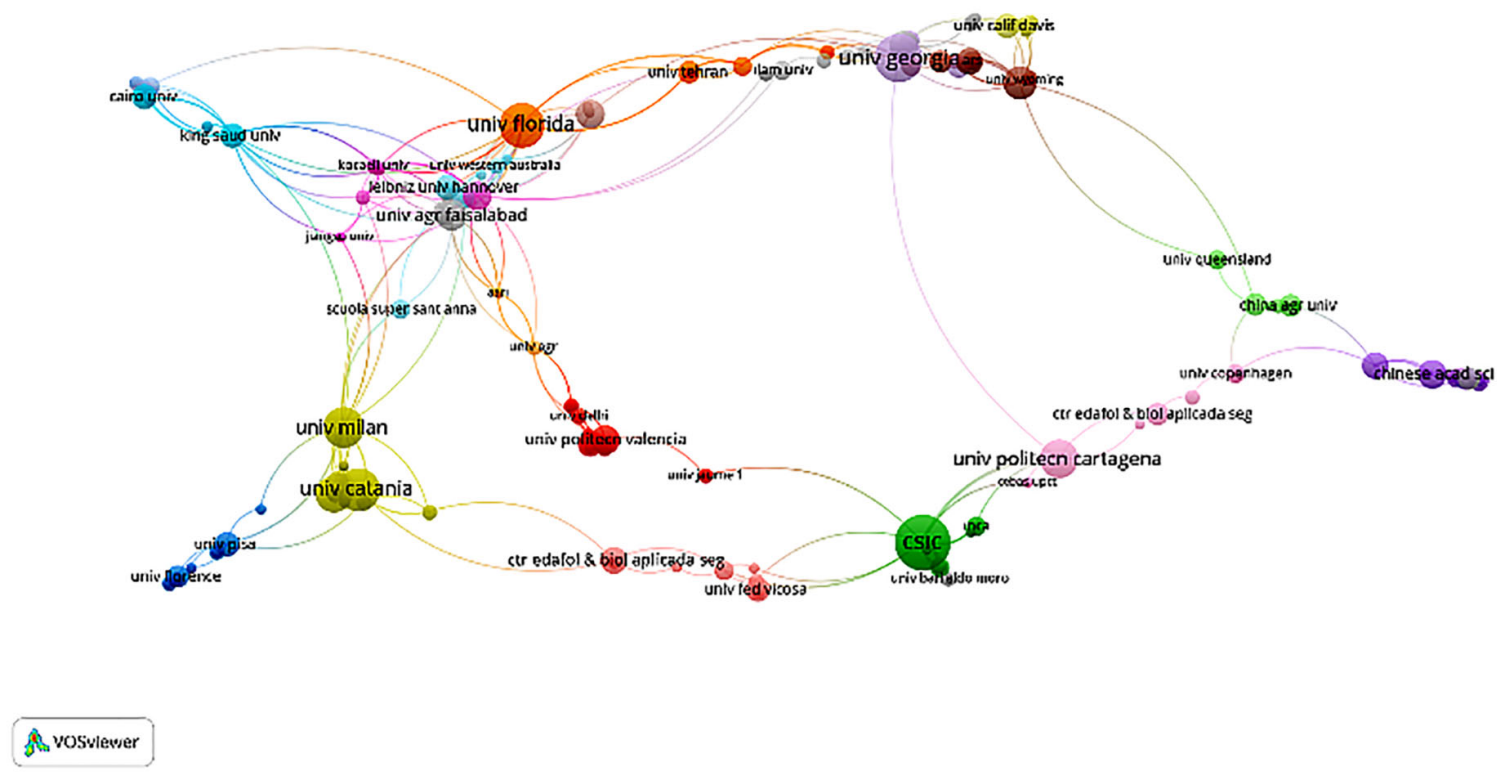
Clustered institutional collaboration network (1995-2025). Created by the authors using VOSviewer.

**Figure 8:**
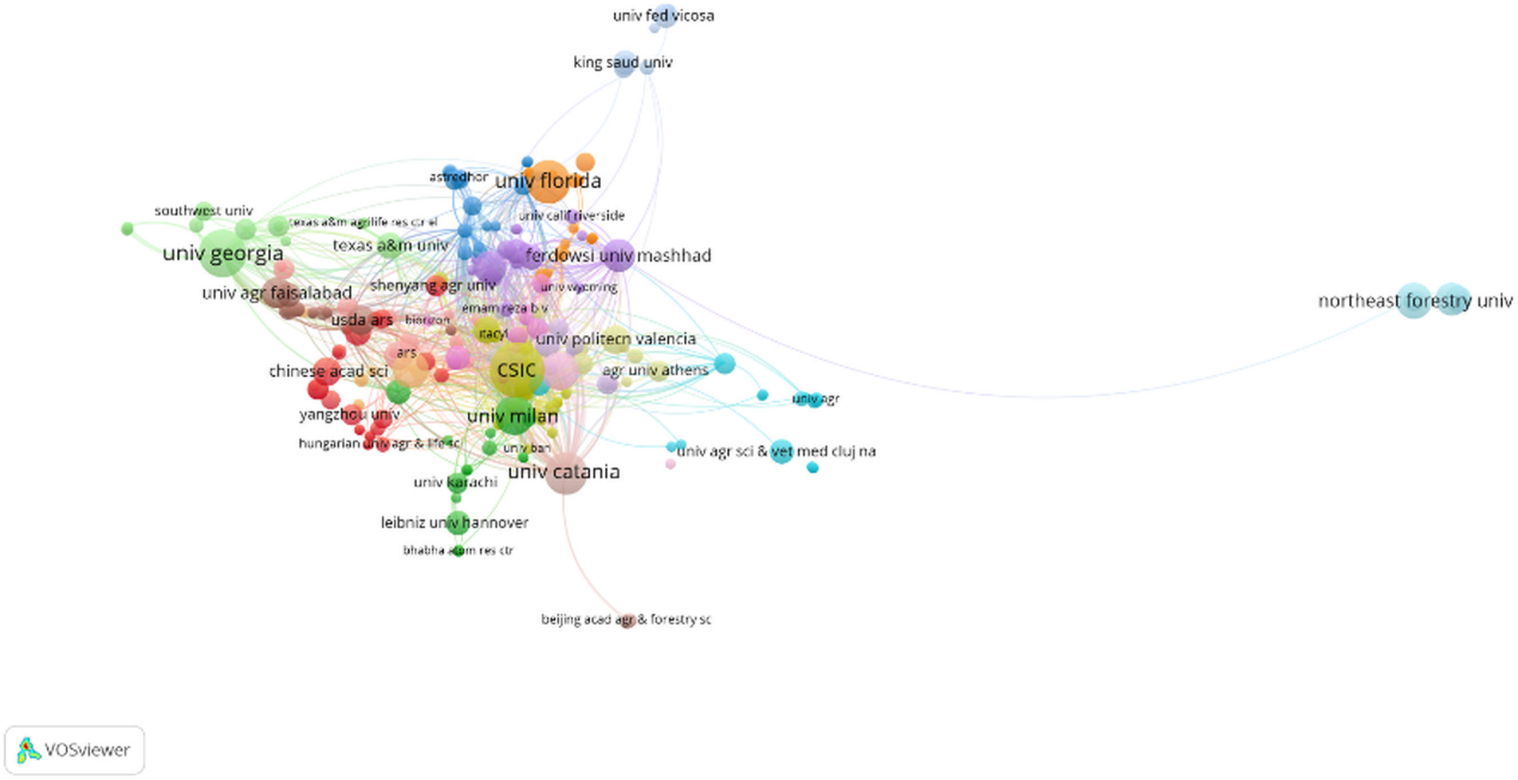
Integrated institutional network (1995–2025) showing key collaboration hubs. Created by the authors using VOSviewer.

Institution-level co-authorship analysis was conducted to examine the structural organization of research collaborations on drought stress in ornamental plants. Two distinct visualizations were generated to reflect different dimensions of institutional connectivity. **Figure 7** presents a cluster-based collaboration map, where institutions are grouped into relatively distinct regional or thematic clusters. Universities such as *University of Florida*, *University of Catania*, and *Chinese Academy of Sciences* appear as central actors within their respective clusters. This structure highlights regionally dominant institutions and reveals discrete networks of cooperation, often shaped by geographical proximity or shared funding mechanisms. In contrast, **Figure 8** displays a more integrated and densely connected network, indicating broader and more complex patterns of collaboration. Institutions such as *CSIC (Spain)*, *University of Milan*, *University of Georgia*, and *Texas A&M University* occupy central positions and demonstrate strong linkages across multiple countries and research hubs. The dense structure of the network reflects the growing globalization of research, with collaborations increasingly crossing regional borders. Taken together, the visualizations provide complementary insights: clustered maps highlight the division of research communities, while the integrated network demonstrates a trend toward globally connected research on drought stress in ornamental plants.

### 3.6 International collaboration patterns in drought-stressed ornamentals

**Figure 9:**
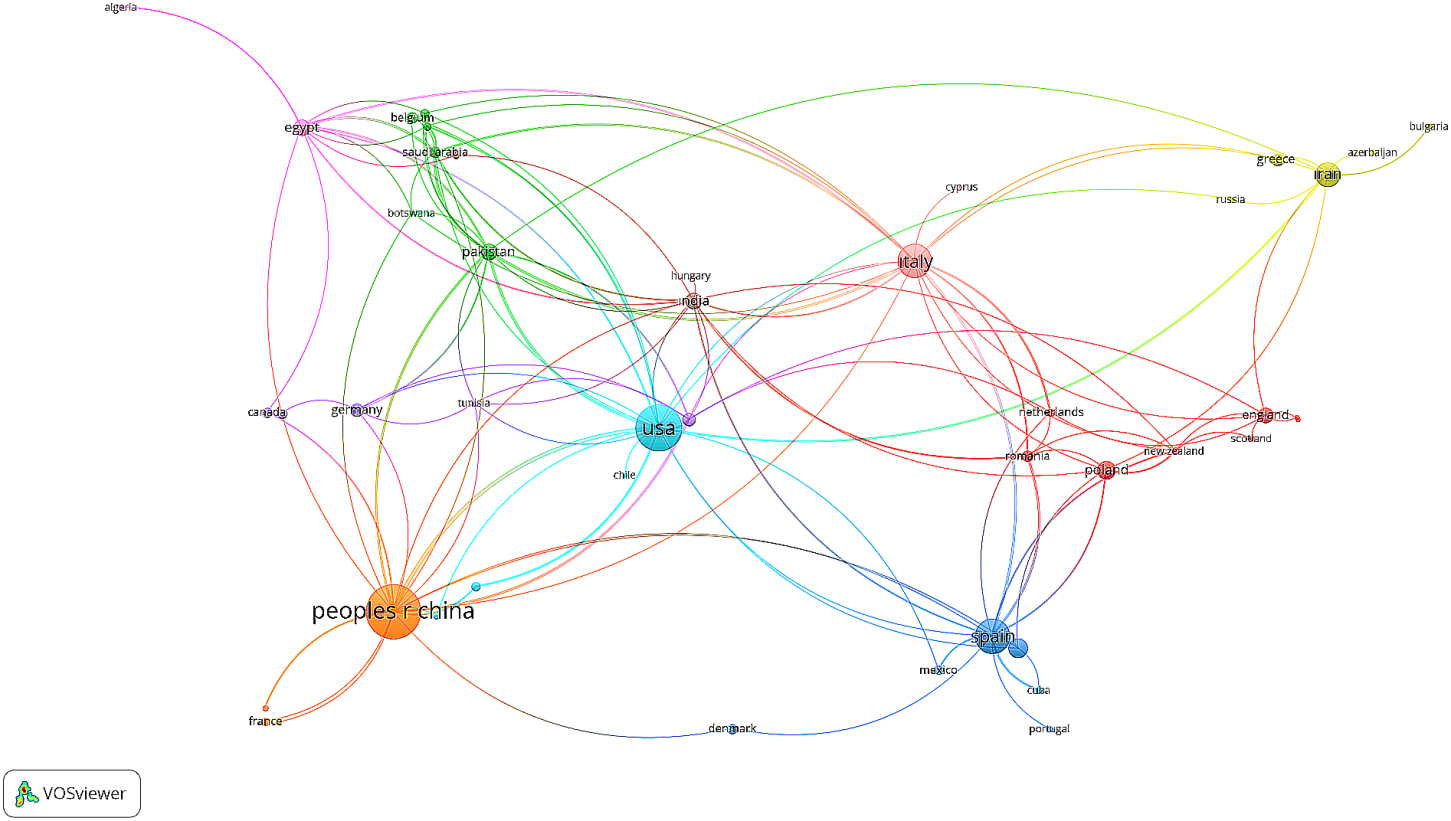
International collaboration map based on Scopus and WoS data (1995–2025). Created by the authors using VOSviewer.

Further analysis of the country co-authorship network revealed a highly collaborative structure, particularly from 2010 onwards. As shown in **Figure 9**, China, the United States, and Spain emerged as the most central countries, forming dense clusters of international collaboration. These countries maintain extensive international collaborations spanning Europe, Asia, and Africa, underscoring their leading role in drought stress research within ornamental horticulture. Other notable contributors include Italy, Iran, and Pakistan, which also exhibited multiple international linkages. The international collaborations observed such as China–France, USA–Chile, and Spain–Mexico—demonstrate that water stress in ornamental horticulture is a shared global challenge.

### 3.7 Citation impact in ornamental drought stress literature

**Figure 10:**
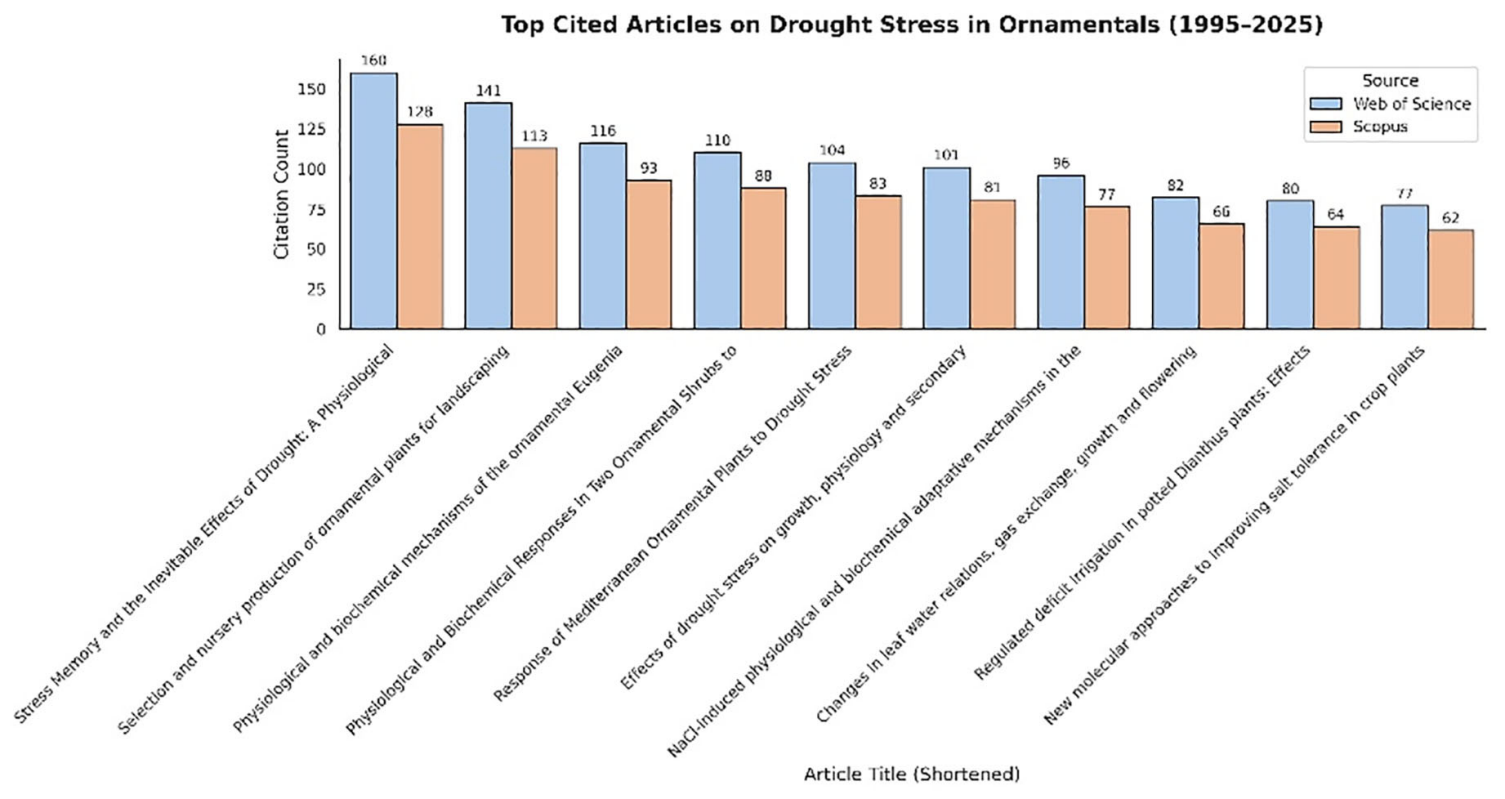
Top cited articles on drought stress in ornamentals (1995–2025).

The citation patterns identified in this study highlight the maturation and concentration of scholarly focus of drought stress research within ornamental horticulture (**Figure 10**). The most frequently cited articles, primarily published in journals such as Frontiers in Plant Science, Scientia Horticulturae, and Journal of Horticultural Science and Biotechnology, consistently address physiological and biochemical adaptation mechanisms to drought conditions. This citation concentration suggests that the academic community places high value on studies elucidating stress-responsive pathways and tolerance traits in ornamental species. Such works not only enhance fundamental understanding but also provide practical insights for nursery selection and landscape management under water-limited environments. An analysis of the most frequently cited articles in the field of drought stress and ornamental plants was conducted using bibliometric data extracted from Web of Science and Scopus for the period 1995–2025. **Figure 10** presents a comparative overview of citation counts for the top 10 most cited publications across both databases. While the ranking of key publications remains largely consistent between the two sources, Web of Science tends to report higher citation values for most documents. This discrepancy may reflect differences in indexing coverage, update frequency, and inclusion criteria between the databases. For example, the article by Fleta-Soriano and Munné-Bosch (2016), addressing drought stress memory in plants, has received over 160 citations in Web of Science, whereas Scopus lists 128 citations, indicating a difference in database coverage. Thematically, highly cited articles predominantly focus on physiological adaptation mechanisms, stress tolerance traits, and biochemical responses of ornamental species under water deficit conditions. These core topics appear to form the conceptual backbone of research in this area, consistently attracting scholarly attention and shaping future inquiry. The citation analysis shows that studies combining practical applications with an understanding of plant responses—particularly in Mediterranean and landscape ornamentals—tend to receive more citations. This highlights the growing importance of climate resilience research in ornamental horticulture, especially in the context of water scarcity and increasing environmental variability.

### 3.8 Citation-based profiling of key contributors in ornamental plant stress studies

**Figure 11:**
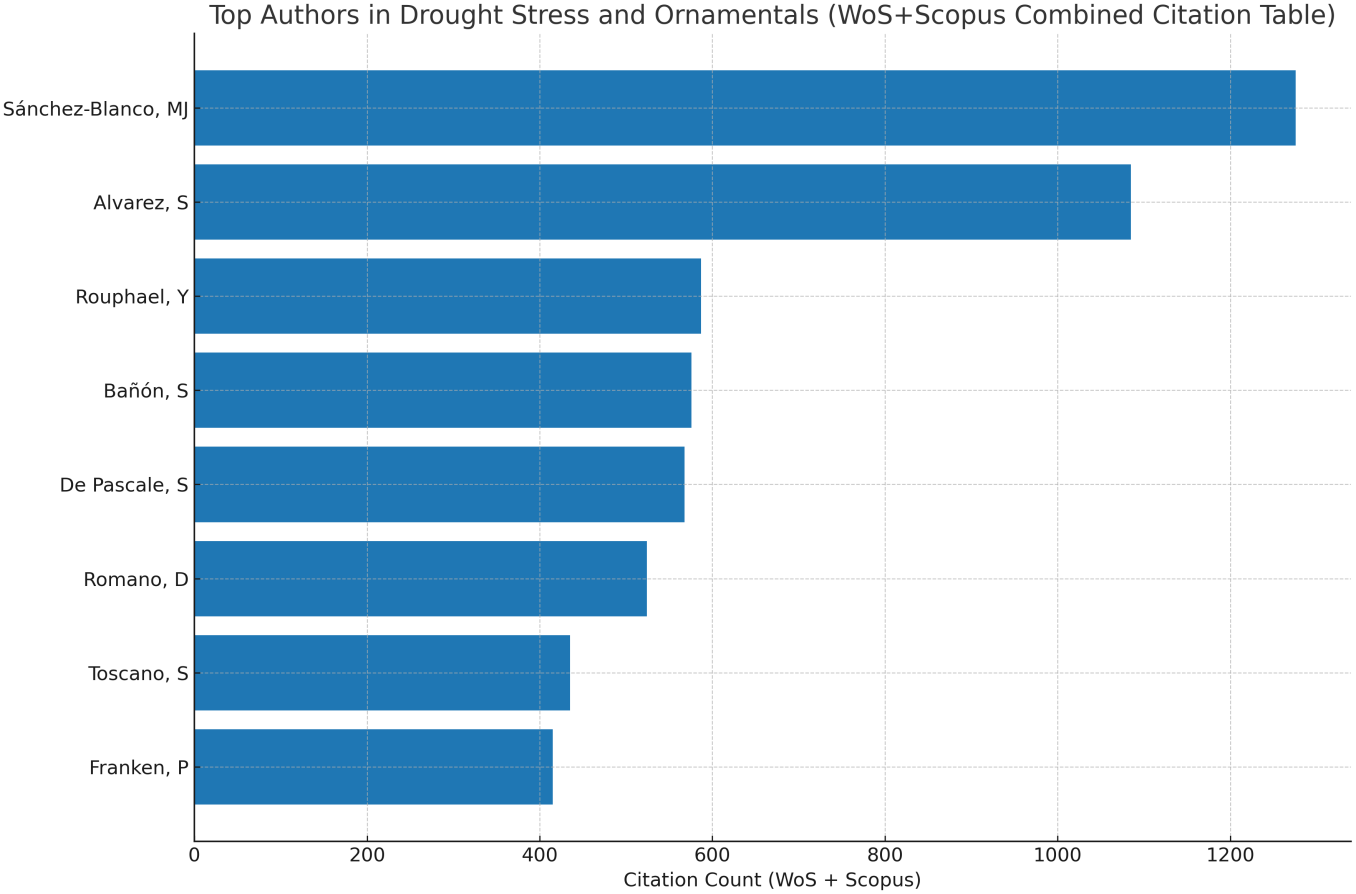
Top authors in drought stress and ornamentals (WoS + Scopus combined citation table).

The citation-based analysis highlights the most frequently cited authors in drought stress research on ornamental plants. As shown in **Figure 11**, the author ranking is based on verified citation counts derived from the combined Web of Science Core Collection and Scopus datasets. Sánchez-Blanco, M.J. and Álvarez, S. occupy the top positions in the citation ranking, while other authors such as Rouphael, Y., Bañón, S., De Pascale, S., Romano, D., Toscano, S., and Franken, P. are also included among the highly cited contributors. Overall, **Figure 11** provides an overview of the citation impact of key authors in this research field.

## 4 Discussion: advances in ornamental plant responses to drought stress

The bibliometric mapping reveals key patterns in the development, intensity, and thematic evolution of drought stress research in ornamental plants over the past three decades. Journals such as *HortScience*, *Scientia Horticulturae*, and *Frontiers in Plant Science* have emerged as primary publication venues, reflecting their alignment with experimental and applied horticultural research. Their bibliographic coupling suggests a shared emphasis on physiological adaptation and drought mitigation strategies. Authorship and institutional data highlight a relatively consolidated academic network, with researchers such as van Iersel, Niu, and Sun playing central roles in advancing the field and fostering international collaboration. These networks are essential for addressing the complex, multi-scale nature of drought stress, which spans molecular biology to ecological applications. The leading role of countries like the USA and China may be attributed to their robust research infrastructure and funding capacity, while active participation from European and Asian institutions signals a growing recognition of drought resilience as a global research priority. Keyword co-occurrence analysis revealed two dominant thematic clusters: one grounded in classical physiological parameters (e.g., stomatal conductance, photosynthesis, proline) and another reflecting emerging molecular themes (e.g., transcription factors, RNA-seq, proteomics). This shift aligns with broader developments in plant stress genomics and reflects a transition from traditional screening methods toward molecular and integrative approaches (Zhao et al., 2024; Chachar et al., 2025). Prominent studies, such as Liu et al. (2011), have underscored the role of antioxidant systems and osmotic regulation in drought tolerance. Likewise, Toscano et al. (2016) demonstrated that differential antioxidant responses in *Photinia fraseri* and *Eugenia uniflora* can assist in selecting drought-adaptive varieties for Mediterranean conditions. These findings support the use of physiological markers in both research and breeding contexts. Coupling analysis further revealed that *Scientia Horticulturae*, *Frontiers in Plant Science*, and *HortScience* consistently host the most cited articles, indicating strong visibility and thematic consistency in the field. Collaborative networks, particularly those anchored by Sun Youping and Niu Genhua, enhance knowledge exchange across borders, though some clusters (e.g., those centered around van Iersel or Chen Jie) remain relatively isolated, suggesting opportunities for greater interdisciplinary integration. Institutions like CSIC, University of Milan, and University of Florida serve as international collaboration hubs. Their centrality in co-authorship networks demonstrates the importance of cross-border alliances in tackling climate-driven challenges in ornamental horticulture. Citation analysis identified Álvarez, Toscano and Sánchez-Blanco as highly influential contributors, largely due to their focus on regulated deficit irrigation and physiological plasticity in Mediterranean ornamentals (Álvarez et al., 2009; Sánchez-Blanco et al., 2009; Toscano et al., 2016). These authors exemplify how region-specific challenges can shape impactful research agendas. Compared to staple crops, ornamental plant research remains less connected globally, with clusters heavily concentrated in Southern Europe. This reflects the Mediterranean region’s vulnerability to drought and a heightened demand for resilient ornamental cultivars (Álvarez and Sánchez-Blanco, 2013; Toscano et al., 2016). While molecular methods are increasingly cited, their implementation remains limited compared to food crops. Studies on stress memory (Fleta-Soriano and Munné-Bosch, 2016) and nursery selection for resilience (Franco et al., 2006) continue to shape the field’s long-term trajectory. Finally, the increasing appearance of terms related to genomics and biotechnology alongside references to applied practices like regulated irrigation and urban landscaping suggests a growing convergence of molecular research with real-world horticultural needs. As Mircea et al. (2024) propose, integrating genetic tools, microbial consortia, and remote sensing technologies could help overcome current limitations and foster a more holistic understanding of drought adaptation in ornamentals.

### 4.1 Research foundations and molecular advances

The evolution of drought stress research in ornamental horticulture has transitioned from classical physiological assessments to more integrated molecular frameworks. Early studies primarily focused on parameters such as water-use efficiency, leaf gas exchange, and visible stress symptoms, which still form the foundation of experimental evaluations. For instance, Cicevan et al. (2016) conducted a comprehensive screening for drought tolerance in Tagetes cultivars based on morphological and physiological traits, underscoring the importance of varietal selection for ornamental plant resilience. However, recent research has increasingly embraced omics-based approaches including transcriptomics, proteomics, and metabolomics to uncover deeper regulatory mechanisms underlying stress responses. Fleta-Soriano and Munné-Bosch (2016) highlighted the concept of stress memory and gene priming in drought adaptation, which has since been supported by transcriptome-wide analyses in ornamental species. Similarly, Sánchez-Blanco et al. (2009) emphasized the importance of osmotic adjustment and antioxidant enzyme activities in conferring drought tolerance in potted ornamental plants, providing early evidence of physiological plasticity under water-deficit conditions. Recent omics approaches further advanced this perspective. In *Iris germanica*, transcriptome profiling revealed over 31,000 differentially expressed genes in response to PEG-induced stress, indicating tissue-specific transcriptional reprogramming (Zhang et al., 2021a). In *Hemerocallis middendorfii*, drought stress triggered hormonal signal transduction and the activation of multiple transcription factor (TF) families such as AP2/ERF, WRKY, MYB, NAC, and bZIP (Qian et al., 2025). Genome-wide expression profiling in *Rosa chinensis* identified key transcriptional regulators such as WRKY, MYB, ERF, and bHLH, alongside MAPK and calcium-mediated signaling cascades (Li et al., 2024). Additionally, multi-omics and transcriptomic approaches in *Dendrobium* species demonstrated genotype-specific antioxidant defenses and transcriptional dynamics under drought conditions (Yuan et al., 2024; Huang et al., 2023). These findings underscore the growing research focus not only on morphological and physiological adaptability but also on the molecular signaling networks and transcriptional regulators that mediate drought tolerance in ornamentals. Moreover, additional ornament species beyond the commonly studied taxa have recently been subjected to transcriptome-based drought stress analysis. For example, *Heimia myrtifolia* exhibited distinct gene expression profiles under water limitation, offering insight into regulatory adaptations in non-model ornamentals (Lin et al., 2022). Transcriptome profiling of *Bombax ceiba* a species valued in ornamental and medicinal contexts identified hundreds of genes with ≥1,000-fold differential expression, including those involved in ubiquitin-mediated proteolysis and oxidative phosphorylation (Zhou et al., 2015). Additionally, studies on *Veronica nakaiana* provide one of the first transcriptome-based drought analyses in a high-altitude ornamental species, emphasizing its molecular adaptability (Kaur et al., 2024). Even in *Helianthus* (sunflower), which also serves aesthetic purposes, transcriptomic investigations have linked physiological recovery after drought to specific stress responsive networks (Janzen et al., 2023; Shen et al., 2023). For instance, *Paeonia ostii* and *Antirrhinum majus* exhibit significant reductions in net photosynthetic rate, stomatal conductance, and chlorophyll fluorescence under prolonged water deficit, coupled with notable decreases in chlorophyll a and b content. In Bougainvillea and Rosa chinensis, drought induces pronounced oxidative stress, reflected in elevated reactive oxygen species (ROS) accumulation, alongside increased activities of antioxidant enzymes such as superoxide dismutase (SOD) and catalase (CAT) (Giordano et al., 2021). Species-specific differences in recovery patterns have also been reported; while some taxa rapidly restore photosynthetic performance upon rewatering, others maintain prolonged physiological impairment (Giordano et al., 2021). Notably, country-level trends show distinct research emphases: China has demonstrated strong leadership in molecular and genomic investigations, particularly in integrating transcriptomics with physiological assays, while the United States has contributed substantially to applied horticultural practices and drought management strategies. In contrast, Spain’s output is characterized by an intermediate approach, combining applied experimentation with emerging molecular techniques. Despite these advances, the application of integrative molecular methods remains largely confined to a small group of commercially important genera, most notably *Petunia hybrida*, *Chrysanthemum morifolium*, *Limonium sinuatum*, *Helianthus annuus*, and *Rosa chinensis* (Wang et al., 2018; González-Orenga et al., 2019; Fu et al., 2021; Park et al., 2021; Cai et al., 2025). Addressing this imbalance through targeted omics-based research in underrepresented yet horticulturally valuable taxa could accelerate the development of drought-resilient ornamental cultivars. Our bibliometric mapping (**Figure 4**) reveals that research in this field has largely focused on physiological and biochemical mechanisms. In particular, increases in antioxidant enzyme activity, proline accumulation, and changes in chlorophyll content under drought conditions are among the most frequently addressed topics. In contrast, studies on morphological traits such as leaf morphology and root development, as well as on molecular-level regulatory mechanisms, remain relatively limited. This highlights the need for wider adoption of transcriptomic, proteomic, and other omics-based approaches. Applying such molecular methods to underrepresented yet horticulturally important ornamental species could enhance our understanding of drought adaptation. In doing so, it may be possible to identify critical pathways in gene expression and signal transduction, ultimately facilitating the development of more resilient cultivars. While traditional physiological and biochemical studies have provided valuable insights to date, complementing them with advanced molecular techniques will likely accelerate scientific progress and broaden the scope of practical applications. To complement the visual representation in **Figure 4**, **Table 3** summarizes the ornamental plant species most frequently investigated under drought stress during the past three decades. For each species, the table outlines the primary physiological and/or molecular response mechanisms documented in the literature, along with representative references.

**Table 3:** Integrated view of drought-stress research foci in ornamental plants (1995–2025): VOSviewer label alignment, physiological/molecular focus, concise findings, and references.

Figure label / species or topic	Dominant process (Physiol./molecular)	Concise finding / focus	Representative references
*Petunia hybrida*	Physiol. + Molecular	Osmotic adjustment, antioxidant responses; ABA-associated regulation under water deficit	Wang et al. (2018)
*Helianthus annuus*	Physiol. + Molecular	Recovery of photosynthesis and carbon metabolism after drought; hormone signaling reprogramming	Janzen et al. (2023); Shen et al. (2023)
*Chrysanthemum morifolium*	Physiol. + Molecular	Photosynthetic performance, ROS detox; TFs and stress-responsive genes implicated	González-Orenga et al. (2019); Giordano et al. (2021)
*Limonium sinuatum*	Physiol. + Molecular	Osmotic adjustment and ion homeostasis under water/salt deficit; ABA-related responses	Fu et al. (2021)
*Rosa chinensis*	Molecular + Physiol.	Transcriptome: WRKY, MYB, ERF, bHLH; MAPK and Ca2+ signaling; antioxidant enzymes under drought	Li et al. (2024); Giordano et al. (2021)
*Heimia myrtifolia*	Molecular	Drought-induced gene expression shifts in a non-model ornamental; regulatory adaptation signals	Lin et al. (2022)
*Bombax ceiba*	Molecular	High-fold DEGs; ubiquitin-mediated proteolysis and oxidative phosphorylation highlighted	Zhou et al. (2015)
*Veronica nakaiana*	Molecular	Transcriptomic drought analysis in a high-altitude ornamental; osmolyte and ABA pathways	Kaur et al. (2024)
*Salvia* sp*lendens*	Physiol.	Antioxidant enzyme activity and delayed senescence patterns under water deficit	Giordano et al. (2021)
*Orchidaceae* spp. *(e.g., Dendrobium)*	Molecular + Physiol.	Genotype-specific antioxidant defense; secondary metabolism and transcriptional dynamics	Yuan et al. (2024); Huang et al. (2023)
*Paeonia ostii*	Physiol. + Molecular	Reductions in photosynthesis, gs, Fv/Fm; DEGs in proline/flavonoid biosynthesis under drought	Giordano et al. (2021)
*Antirrhinum majus*	Physiol.	Decline in chlorophyll a/b; increased ROS; antioxidant responses	Giordano et al. (2021)
*Bougainvillea* spp.	Physiol.	Enhanced SOD, CAT; membrane stability under drought; quality maintenance	Giordano et al. (2021)
Antioxidant enzymes (SOD, CAT, POD)	Physiol./Biochem.	Most frequent physiological theme; ROS scavenging correlates with tolerance and quality retention	Sánchez-Blanco et al. (2009); Giordano et al. (2021)
Proline / Osmotic adjustment	Physiol./Biochem.	Accumulation relates to osmoprotection; often co-reported with antioxidant activity	Sánchez-Blanco et al. (2009)
Chlorophyll & PSII efficiency (Fv/Fm)	Physiol.	Widely used stress index; drops under severe drought; recovery varies by species	Giordano et al. (2021)
ABA / Hormonal signaling	Molecular	Central in stomatal closure and stress gene activation; interacts with GA/auxin pathways	Qian et al. (2025); Giordano et al. (2021)
Transcription factors (WRKY, MYB, NAC, ERF, bZIP)	Molecular	Core regulators enriched in drought-responsive transcriptomes of ornamentals	Li et al. (2024); Qian et al. (2025)
Stress memory / priming	Concept / Molecular	Evidence for priming mechanisms shaping recurrent drought responses	Fleta-Soriano and Munné-Bosch (2016)
General physiological screens (e.g., Tagetes)	Physiol.	Early varietal screening via gas exchange, WUE, visual traits informs selection	Cicevan et al. (2016)

### 4.2 Regional disparities and collaborative gaps

While the volume of scientific output on drought stress in ornamental plants has steadily expanded over the past three decades, regional disparities remain evident. Most high-impact publications originate from institutions in China, the United States, Spain, and Italy countries with well-established research infrastructures and robust funding schemes for plant stress physiology and horticulture (Toscano et al., 2016; Chachar et al., 2025). In contrast, contributions from Southeast Asia and South America are comparatively fewer and tend to remain peripheral within the international co-authorship and institutional networks (**Figures 7**, **8**). This imbalance potentially limits the transferability of existing findings to other agro-climatic contexts, particularly those facing severe drought conditions. For example, countries in tropical and subtropical zones many of which are highly vulnerable to climate-induced water scarcity remain underrepresented in leading collaborative clusters, despite growing ornamental markets and ecological stress pressures in these regions (Álvarez et al., 2009; Jafari et al., 2019). Strengthening cross-regional cooperation particularly through multilateral consortia and institutional twinning programs could promote the inclusion of more diverse taxa, genotypes, and production systems in future studies. Moreover, encouraging open-access publishing and multilingual dissemination platforms may help improve visibility and citation potential for researchers in lower-representation regions (Franco et al., 2006; Fleta-Soriano and Munné-Bosch, 2016).

### 4.3 Journal coupling and publication strategy

The bibliographic coupling analysis (**Figure 5**) indicates that drought-related ornamental research is concentrated within a narrow range of horticultural journals, notably *Scientia Horticulturae*, *Frontiers in Plant Science*, and *HortScience*. While these journals offer focused visibility, they may also restrict interdisciplinary outreach, particularly toward climate science, environmental modeling, or landscape ecology communities. Highly cited articles such as Franco et al. (2006) and Toscano et al. (2016) succeeded not only through content quality but also by selecting platforms with strong field-specific relevance and international readership. Thus, journal selection emerges as a strategic factor influencing citation performance and thematic diffusion. To enhance both visibility and scholarly impact, future authors might consider targeting journals with broader ecological or environmental scopes—such as *Environmental and Experimental Botany* or *Agricultural Water Management* while maintaining methodological rigor aligned with horticultural standards.

### 4.4 Methodological considerations and risk of bias

As this study is based on secondary bibliometric data retrieved from established scientific databases (Scopus and Web of Science), traditional sources of bias such as selection bias, performance bias, or reporting bias are not applicable. However, certain limitations may influence the comprehensiveness and neutrality of the findings. These include database coverage discrepancies, indexing delays, language bias (favoring English-language publications), and the underrepresentation of regional or non-indexed journals. Furthermore, the exclusion of unpublished materials, institutional reports, conference abstracts, and other non-peer-reviewed documents may have limited the breadth of the review by omitting potentially relevant sources not indexed in major databases. Although efforts were made to harmonize and clean the dataset manually, metadata inconsistencies may still introduce minor distortions in network visualizations or keyword clustering. Nevertheless, the transparent reporting of search strategy, inclusion criteria, and PRISMA-aligned study selection workflow enhances the reliability and reproducibility of the analysis.

## 5 Conclusion: future directions in drought-resilient ornamental horticulture

This study highlights the growing scientific interest in drought stress research within ornamental horticulture. The findings indicate a shift from classical physiological approaches to more integrated molecular and multidisciplinary strategies. While significant progress has been made, particularly in countries with strong research capacity, gaps remain in regional representation and species diversity. Strengthening international collaborations and expanding research on underexplored ornamental species will be essential for developing more resilient and adaptive practices in the face of increasing water scarcity.

## Data Availability

All bibliometric data were retrieved from [Scopus/Web of Science]. Processed datasets are available from the corresponding author on reasonable request.

## References

[B1] Álvarez S. Navarro A. Bañón S. Sánchez-Blanco M. J. (2009). Regulated deficit irrigation in potted Dianthus plants: Effects of severe and moderate water stress on growth and physiological responses. Sci. Hortic. 122, 579–585. doi: 10.1016/j.scienta.2009.06.030, PMID: 41727822

[B2] Álvarez S. Sánchez-Blanco M. J. (2013). Changes in growth rate, root morphology and water use efficiency of potted *Callistemon citrinus* plants in response to different levels of water deficit. Sci. Hortic. 156, 54–62. doi: 10.1016/j.scienta.2013.03.024, PMID: 41727822

[B3] Anjum S. A. Xie X. Y. Wang L. C. Saleem M. F. Man C. Lei W. (2011). Morphological, physiological and biochemical responses of plants to drought stress. Afr. J. Agric. Res. 6, 2026–2032. doi: 10.5897/AJAR10.027, PMID: 38147025

[B4] Cai li Bi Zhu Z. Tian Q. Liu T. Wang H. J. (2025). The effects of drought stress on seed germination and seedling physiology of three limonium species. bioRxiv 2025, 36 pages. doi: 10.2139/ssrn.5045450, PMID: 40330906

[B5] Chachar S. Ahmed N. Hu X. (2025). Future-proofing ornamental plants: Cutting-edge strategies for drought resistance and sustainability. Physiol. Plant 177, e70255. doi: 10.1111/ppl.70255, PMID: 40325615

[B6] Cicevan R. Al Hassan M. Sestras A. F. Prohens J. Vicente O. Sestras R. E. . (2016). Screening for drought tolerance in cultivars of the ornamental genus Tagetes (Asteraceae). PeerJ 4, e2133. doi: 10.7717/peerj.2133, PMID: 27326384 PMC4911946

[B7] Cui Z. Huang H. Du T. Chen J. Huang S. Dai Q. (2023). Integrated transcriptome and metabolome revealed the drought responsive metabolic pathways in Oriental Lily (*Lilium* L.). PeerJ 11, e16658. doi: 10.7717/peerj.16658, PMID: 38130923 PMC10734436

[B8] Cui Y. Ouyang S. Zhao Y. Tie L. Shao C. Duan H. (2022). Plant responses to high temperature and drought: A bibliometrics analysis. Front. Plant Sci. 13. doi: 10.3389/fpls.2022.1052660, PMID: 36438139 PMC9681914

[B9] Fleta-Soriano E. Munné-Bosch S. (2016). Stress memory and the inevitable effects of drought: a physiological perspective. Front. Plant Sci. 7, 143. doi: 10.3389/fpls.2016.00143, PMID: 26913046 PMC4753297

[B10] Franco J. A. Martínez-Sánchez J. J. Fernández J. A. Bañón S. (2006). Selection and nursery production of ornamental plants for landscaping and xerogardening in semi-arid environments. J. Hortic. Sci. Biotechnol. 81, 3–17. doi: 10.1080/14620316.2006.11512022, PMID: 41669619

[B11] Fu H. Zeng T. Zhao Y. Luo T. Deng H. Meng C. . (2021). Identification of chlorophyll metabolism-and photosynthesis-related genes regulating green flower color in chrysanthemum by integrative transcriptome and weighted correlation network analyses. Genes 12, 449. doi: 10.3390/genes12030449, PMID: 33801035 PMC8004015

[B12] Giordano M. Petropoulos S. A. Cirillo C. Rouphael Y. (2021). Biochemical, physiological, and molecular aspects of ornamental plants adaptation to deficit irrigation. Horticulturae 7, 107. doi: 10.3390/horticulturae7050107, PMID: 41725453

[B13] González-Orenga S. Al Hassan M. Llinares J. V. Lisón P. López-Gresa M. P. Verdeguer M. . (2019). Qualitative and quantitative differences in osmolytes accumulation and antioxidant activities in response to water deficit in four Mediterranean Limonium species. Plants 8, 506. doi: 10.3390/plants8110506, PMID: 31731597 PMC6918351

[B14] Huang H. Jiao Y. Tong Y. Wang Y. (2023). Comparative analysis of drought-responsive biochemical and transcriptomic mechanisms in two *Dendrobium officinale* genotypes. Ind. Crops Prod. 199, 116766. doi: 10.1016/j.indcrop.2023.116766, PMID: 41727822

[B15] IPCC (2021). Intergovernmental Panel on Climate Change. Climate Change 2021: The physical science basis. contribution of working group ı to the sixth assessment report of the IPCC (Geneva, Switzerland: Cambridge University Press).

[B16] Jafari S. Garmdareh S. E. H. Azadegan B. (2019). Effects of drought stress on morphological, physiological, and biochemical characteristics of stock plant (*Matthiola incana* L.). Sci. Hortic. 253, 128–133. doi: 10.1016/j.scienta.2019.04.033, PMID: 41727822

[B17] Janzen G. M. Dittmar E. L. Langlade N. B. Blanchet N. Donovan L. A. Temme A. A. . (2023). Similar transcriptomic responses to early and late drought stresses produce divergent phenotypes in sunflower (*Helianthus annuus* L.). Int. J. Mol. Sci. 24, 9351. doi: 10.3390/ijms24119351, PMID: 37298305 PMC10253505

[B18] Kapoor D. Bhardwaj S. Landi M. Sharma A. Ramakrishnan M. Sharma A. . (2020). The impact of drought in plant metabolism: how to exploit tolerance mechanisms to increase crop production. Appl. Sci. 10, 5692. doi: 10.3390/app10165692, PMID: 41725453

[B19] Kaur C. Kwon Y. H. Song H. Y. Gil M. Rhie Y. H. Lee G. J. (2024). Unraveling the adaptive mechanisms of Veronica nakaiana in response to drought stress: A transcriptome-based study. Sci. Hortic. 338, 113799. doi: 10.1016/j.scienta.2024.113799, PMID: 41727822

[B20] Kittipornkul P. Treesubsuntorn C. Kobthong S. Yingchutrakul Y. Julpanwattana P. Thiravetyan P. (2024). The potential of proline as a key metabolite to design real-time plant water deficit and low-light stress detector in ornamental plants. Environ. Sci. pollut. Res. 31, 36152–36162. doi: 10.1007/s11356-023-27990-3, PMID: 37284956

[B21] Li S. Xu J. Cao Y. Wu J. Liu Q. Zhang D. (2024). Genome-Wide Analyses of CCHC Family Genes and Their Expression Profiles under Drought Stress in Rose (*Rosa chinensis*). Int. J. Mol. Sci. 25, 8983. doi: 10.3390/ijms25168983, PMID: 39201669 PMC11354476

[B22] Lin L. Wang J. Wang Q. Ji M. Hong S. Shang L. . (2022). Transcriptome approach reveals the response mechanism of Heimia myrtifolia (Lythraceae, Myrtales) to drought stress. Front. Plant Sci. 13, 877913. doi: 10.3389/fpls.2022.877913, PMID: 35874015 PMC9305661

[B23] Liu C. Liu Y. Guo K. Fan D. Li G. Zheng Y. . (2011). Effect of drought on pigments, osmotic adjustment and antioxidant enzymes in six woody plant species in karst habitats of southwestern China. Environ. Exp. Bot. 71, 174–183. doi: 10.1016/j.envexpbot.2010.11.012, PMID: 41727822

[B24] Mircea D. M. Boscaiu M. Sestras R. E. Sestras A. F. Vicente O. (2024). Abiotic stress tolerance and invasive potential of ornamental plants in the Mediterranean area: implications for sustainable landscaping. Agronomy 15, 52. doi: 10.3390/agronomy15010052, PMID: 41725453

[B25] Molina M. O. Soares P. M. M. Lima M. M. Gaspar T. H. Lima D. C. A. Ramos A. M. . (2025). Updated insights on climate change-driven temperature variability across historical and future periods. Clim. Change 178, 97. doi: 10.1007/s10584-025-03937-0, PMID: 41728210

[B26] Naresh R. Tomar P. Singh R. K. (2024). “ State of the art of omics technologies in ornamental plant research,” in *Ornam. Hortic.:* Latest Cultivation Practices and Breeding Technologies, (Singapore: Springer) 175–191.

[B27] Page M. J. McKenzie J. E. Bossuyt P. M. Boutron I. Hoffmann T. C. Mulrow C. D. . (2021). The PRISMA 2020 statement: an updated guideline for reporting systematic reviews. BMJ 372, n71. doi: 10.1136/bmj.n71, PMID: 33782057 PMC8005924

[B28] Park S. Wijeratne A. J. Moon Y. Waterland N. L. (2021). Time-course transcriptomic analysis of *Petunia× hybrida* leaves under water deficit stress using RNA sequencing. PloS One 16, e0250284. doi: 10.1371/journal.pone.0250284, PMID: 33901201 PMC8075263

[B29] Qian Y. Yu H. Lu S. Bai Y. Meng Y. Chen L. . (2025). Transcriptome analysis reveals the role of plant hormone signal transduction pathways in the drought stress response of Hemerocallis middendorffii. Plants 14, 1082. doi: 10.3390/plants14071082, PMID: 40219150 PMC11991170

[B30] Rebi A. Ejaz I. Khatana M. A. Alvi A. B. A. Irfan M. Wang G. . (2024). Effect of irrigation levels on the physiological responses of petunia cultivars for selection. Ecol. Front. 44, 206–216. doi: 10.1016/j.chnaes.2023.12.001, PMID: 41727822

[B31] Sahithi B. M. Razi K. Al Murad M. Vinothkumar A. Jagadeesan S. Benjamin L. K. . (2021). Comparative physiological and proteomic analysis deciphering tolerance and homeostatic signaling pathways in chrysanthemum under drought stress. Physiol. Plant 172, 289–303. doi: 10.1111/ppl.13142, PMID: 32459861

[B32] Sánchez-Blanco M. J. Álvarez S. Navarro A. Bañón S. (2009). Changes in leaf water relations, gas exchange, growth and flowering quality in potted geranium plants irrigated with different water regimes. J. Plant Physiol. 166, 467–476. doi: 10.1016/j.jplph.2008.06.015, PMID: 18778872

[B33] Sánchez-Blanco M. J. Ortuño M. F. Bañon S. Álvarez S. (2019). Deficit irrigation as a strategy to control growth in ornamental plants and enhance their ability to adapt to drought conditions. J. Hortic. Sci. Biotechnol. 94, 137–150. doi: 10.1080/14620316.2019.1570353, PMID: 41669619

[B34] Shen J. Wang X. Song H. Wang M. Niu T. Lei H. . (2023). Physiology and transcriptomics highlight the underlying mechanism of sunflower responses to drought stress and rehydration. Iscience 26, 1–18. doi: 10.1016/j.isci.2023.108112, PMID: 37860690 PMC10583116

[B35] Toscano S. Farieri E. Ferrante A. Romano D. (2016). Physiological and biochemical responses in two ornamental shrubs to drought stress. Front. Plant Sci. 7. doi: 10.3389/fpls.2016.00645, PMID: 27242846 PMC4867676

[B36] Toscano S. Ferrante A. Romano D. (2019). Response of Mediterranean ornamental plants to drought stress. Horticulturae 5, 6. doi: 10.3390/horticulturae5010006, PMID: 41725453

[B37] Toscano S. Romano D. (2021). Morphological, physiological, and biochemical responses of zinnia to drought stress. Horticulturae 7, 362. doi: 10.3390/horticulturae7100362, PMID: 41725453

[B38] Wang K. Bai Z. Y. Liang Q. Y. Liu Q. L. Zhang L. Pan Y. Z. . (2018). Transcriptome analysis of chrysanthemum (*Dendranthema grandiflorum*) in response to low temperature stress. BMC Genomics 19, 1–19. doi: 10.1186/s12864-018-4706-x, PMID: 29720105 PMC5930780

[B39] Xiong Y. Qu Y. Han H. Chen F. Li L. Tang H. . (2021). Unraveling physiological and metabolomic responses involved in *Phlox subulata* L. tolerance to drought stress. Plant Mol. Biol. Rep. 39, 98–111. doi: 10.1007/s11105-020-01238-7, PMID: 41728210

[B40] Yuan Y. Zuo J. Wan X. Zhou R. Xing W. Liu S. (2024). Multi-omics profiling reveal responses of three major Dendrobium species from different growth years to medicinal components. Front. Plant Sci. 15, 1333989. doi: 10.3389/fpls.2024.1333989, PMID: 38463561 PMC10920241

[B41] Zhang C. Chen J. Huang W. Song X. Niu J. (2021b). Transcriptomics and metabolomics reveal purine and phenylpropanoid metabolism response to drought stress in *Dendrobium sinense*, an endemic orchid species in Hainan Island. Front. Genet. 12, 692702. doi: 10.3389/fgene.2021.692702, PMID: 34276795 PMC8283770

[B42] Zhang J. Huang D. Zhao X. Zhang M. (2021a). Evaluation of drought resistance and transcriptome analysis for the identification of drought-responsive genes in *Iris germanica*. Sci. Rep. 11, 16308. doi: 10.1038/s41598-021-95633-z, PMID: 34381085 PMC8358056

[B43] Zhao Z. Liu A. Zhang Y. Yang X. Yang S. Zhao K. (2024). Effects of progressive drought stress on the growth, ornamental values, and physiological properties of *Begonia semperflorens*. Horticulturae 10, 405. doi: 10.3390/horticulturae10040405, PMID: 41725453

[B44] Zhou Z. Ma H. Lin K. Zhao Y. Chen Y. Xiong Z. . (2015). RNA-seq reveals complicated transcriptomic responses to drought stress in a nonmodel tropic plant, Bombax ceiba L. Evolutionary Bioinf. 11, EBO–S20620. doi: 10.4137/EBO.S20620, PMID: 26157330 PMC4479181

