# Correction: Blossoms amid drought: a bibliometric mapping of research on drought stress in ornamental plants (1995–2025)

**DOI:** 10.3389/fpls.2025.1745158

**Published:** 2026-01-02

**Authors:** Ümmü Özgül Karagüzel

**Affiliations:** Department of Horticulture, Faculty of Agriculture, Recep Tayyip Erdoğan University, Rize, Türkiye

**Keywords:** drought stress, floriculture, physiological responses, omics, climate-resilient horticulture, collaboration networks flowering

There was a mistake in **Figure 11** and its caption as published. The previous figure displayed an incorrect author ranking order due to an error in the underlying citation counts derived from the VOSviewer table. The previous version was generated based on the VOSviewer citation table using only Web of Science data, which resulted in an incomplete author ranking. The corrected **Figure 11**, based on combined and verified citation counts from the Web of Science Core Collection and Scopus databases, and its caption, appear below.

**Figure 11:**
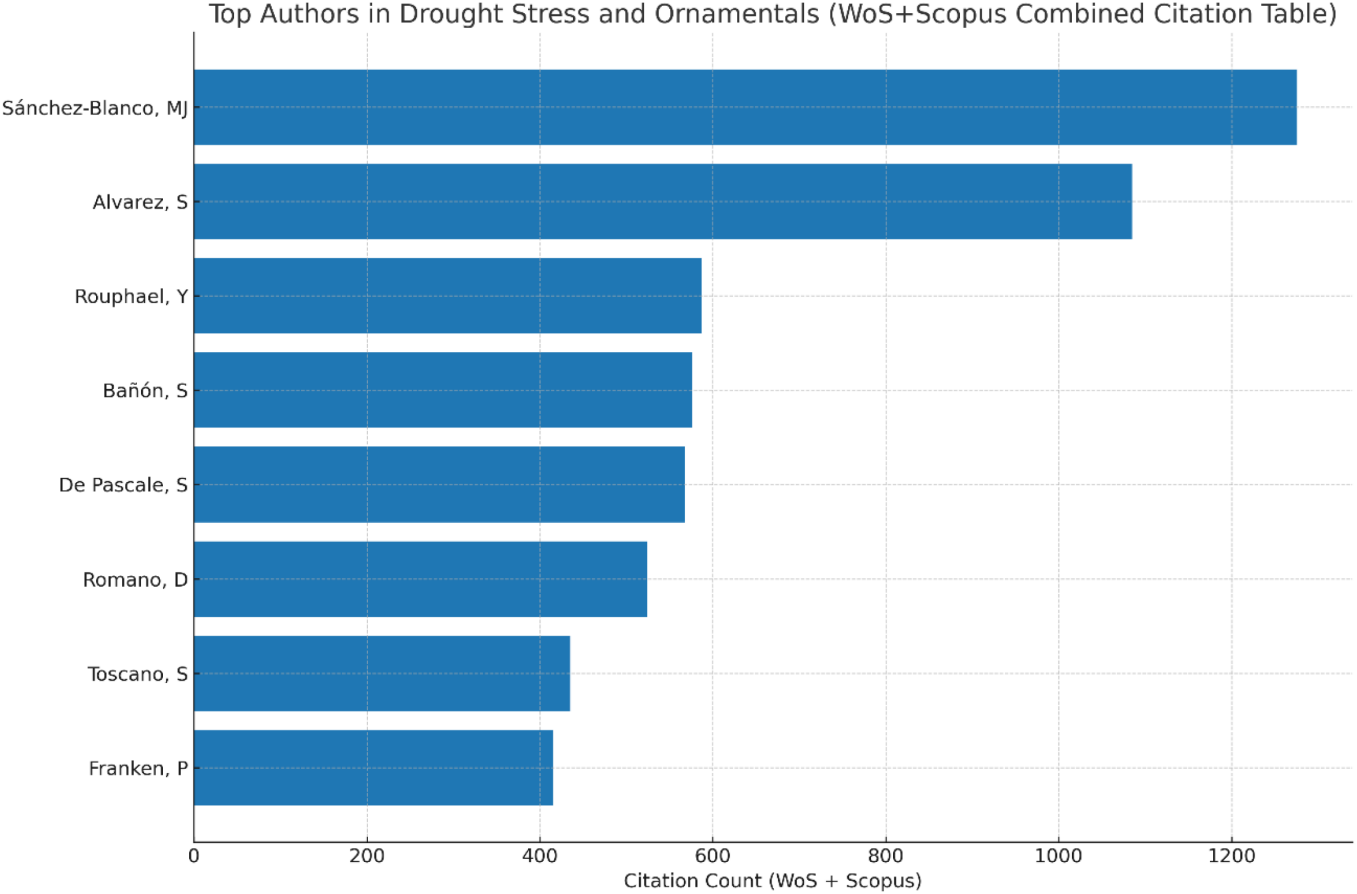
Top authors in drought stress and ornamentals (WoS + Scopus combined citation table).

The original version of this article has been updated.

