# Correction: Blossoms amid drought: a bibliometric mapping of research on drought stress in ornamental plants (1995–2025)

**DOI:** 10.3389/fpls.2026.1799109

**Published:** 2026-02-24

**Authors:** Ümmü Özgül Karagüzel

**Affiliations:** Department of Horticulture, Faculty of Agriculture, Recep Tayyip Erdoğan University, Rize, Türkiye

**Keywords:** drought stress, floriculture, physiological responses, omics, climate-resilient horticulture, collaboration networks flowering

In the published article, the descriptive paragraph in the **Results** section referring to **Figure 11** (Author Citation Impact) was not updated after the Correction that replaced **Figure 11** and its caption.

A correction has been made to the **Results**, *Section 3.8 “Citation-based profiling of key contributors in ornamental plant stress studies”*, paragraph 1.

The incorrect paragraph read as follows, “The citation analysis revealed a distinct pattern of scholarly influence among researchers investigating drought stress in ornamental plants. As shown in **Figure 11**, Sara Álvarez emerged as the most cited author, followed by Stefania Toscano and Daniela Romano, each contributing extensively to the physiological and biochemical understanding of stress tolerance mechanisms. Notably, Sánchez-Blanco et al. (2019) also ranked among the top contributors, reflecting their sustained efforts in Mediterranean species’ resilience studies. The prominence of these authors corresponds closely with institutional clusters in Spain and Italy, reinforcing regional research leadership. This pattern aligns with the broader trend of localized expertise in abiotic stress adaptation within ornamental horticulture. Furthermore, the observed citation impact underscores the centrality of interdisciplinary approaches ranging from field trials to molecular assessments in shaping citation visibility and long-term research relevance. Although authors such as van Iersel Marc W. and Chen Jie occupy central positions within the co-authorship network (**Figure 6**), they do not appear among the top-cited authors in **Figure 11**. This discrepancy may be attributed to their collaborative roles in multi-authored publications or their focus on specialized subtopics within the broader field, which may yield lower overall citation counts despite high connectivity within the research network.”

The correct paragraph should read as, “The citation-based analysis highlights the most frequently cited authors in drought stress research on ornamental plants. As shown in **Figure 11**, the author ranking is based on verified citation counts derived from the combined Web of Science Core Collection and Scopus datasets. Sánchez-Blanco, M.J. and Álvarez, S. occupy the top positions in the citation ranking, while other authors such as Rouphael, Y., Bañón, S., De Pascale, S., Romano, D., Toscano, S., and Franken, P. are also included among the highly cited contributors. Overall, **Figure 11** provides an overview of the citation impact of key authors in this research field.”

The original version of this article has been updated.

